# ProNGF processing in adult rat tissues and bioactivity of NGF prodomain peptides

**DOI:** 10.1002/2211-5463.13768

**Published:** 2024-03-01

**Authors:** Marie Anne Makoudjou, Elena Fico, Pamela Rosso, Viviana Triaca, Lucio De Simone, Daniela Rossetti, Franca Cattani, Marcello Allegretti, Paola Tirassa

**Affiliations:** ^1^ Cellular and Molecular Biology, Department of Biology University of Rome “Tor Vergata” Rome Italy; ^2^ Institute of Biochemistry and Cell Biology (IBBC) National Research Council (CNR) Rome Italy; ^3^ Institute of Biochemistry and Cell Biology (IBBC) National Research Council (CNR) Campus A. Buzzati‐Traverso, Monterotondo Rome Italy; ^4^ Dompé Farmaceutici S.p.A L'Aquila Italy

**Keywords:** apoptosis, inflammation, nerve growth factor, peptides, prodomain, proNGF

## Abstract

The neurotrophin nerve growth factor (NGF) and its precursor proNGF are both bioactive and exert similar or opposite actions depending on the cell target and its milieu. The balance between NGF and proNGF is crucial for cell and tissue homeostasis and it is considered an indicator of pathological conditions. Proteolytical cleavage of proNGF to the mature form results in different fragments, whose function and/or bioactivity is still unclear. The present study was conducted to investigate the distribution of proNGF fragments derived from endogenous cleavage in brain and peripheral tissues of adult rats in the healthy condition and following inflammatory lipopolysaccharide (LPS) challenge. Different anti‐proNGF antibodies were tested and the presence of short peptides corresponding to the prodomain sequence (pdNGFpep) was identified. Processing of proNGF was found to be tissue‐specific and accumulation of pdNGFpeps was found in inflamed tissues, mainly in testis, intestine and heart, suggesting a possible correlation between organ functions and a response to insults and/or injury. The bioactivity of pdNGFpep was also demonstrated *in vitro* by using primary hippocampal neurons. Our study supports a biological function for the NGF precursor prodomain and indicates that short peptides from residues 1–60, differing from the 70–110 sequence, induce apoptosis, thereby opening the way for identification of new molecular targets to study pathological conditions.

## Graphical abstract

The balance and interaction of TrkA/p75NTR with proNGF and its related cleavage product NGF and pdNGFpeps play a pivotal role in the maintenance of structural and functional integrity of target organs. Imbalance of NGF and pdNGFpeps triggered by inflammation as well as deafferentation might activate apoptosis signals and contribute to insurgence and progression of organ damage and disease.

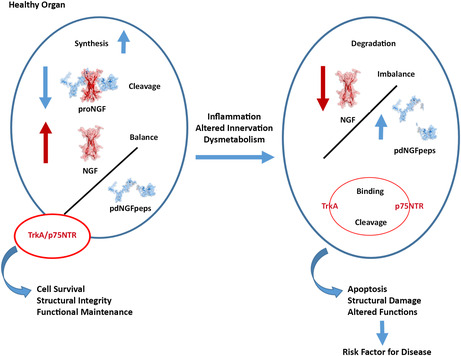

**Abbreviations**
b.w.: body weight
BCA: bicinchoninic acid
BSA: Bovine Serum Albumin
CTRL: control
DAPI: 4′,6‐diamidino‐2‐phenylindole
ECL: Enhanced Chemiluminescence
LPS: lipopolysaccharide
NB: neurobasal medium
NGF: Nerve Growth Factor
p75NTR: p75 Neurotrophin Receptor
pdNGF: NGF prodomain
PdNGFpeps: prodomain peptides of NGF precursor
pJNK: phosphorylated c‐Jun N‐terminal kinase
PMSF: PhenylMethylSulfonyl Fluoride
proNGF: Nerve Growth Factor precursor
RT: room temperature
SD: standard deviation
SEM: standard error of the mean
TrkA: Tropomyosin related Kinase A
WB: Western blot

The neurotrophin Nerve Growth Factor (NGF) is a survival, trophic and differentiating factor, acting on both neuronal and non‐neuronal cells [1, 2, 3], which is almost ubiquitously expressed in the brain and peripheral organs of mammals, including humans [4, 5, 6]. NGF, like other neurotrophins, is initially synthesized as a precursor (proNGF) that is proteolytically cleaved to a biologically active form of about 13kDa (herein indicated as NGF).

From its discovery, and for almost the following 50 years, NGF has been the only one to be recognised as mature “bioactive”, but the evidences that the NGF precursor (proNGF) might also bind the NGF receptors named as p75 Neurotrophin Receptor (p75NTR) and Tropomyosin related Kinase A (TrkA) [7, 8, 9, 10], and exert trophic, even if with less ability than the mature form, as well as proapoptotic effects [11, 12], have definitively changed the idea “on what we thought to know about neurotrophins” [13], and opened a new field of investigation on the role of proNGF and its cleavage in tissues.

Different forms of proNGF have been identified. A glycosylated proNGF form of around 53 kDa, characterised by two glycosylation sites on the NGF prodomain (pdNGF), and on one site on the mature, is reported in several studies [14, 15] but its function is not well understood. The N‐glycosylations at pdNGF site appear as necessary for the proNGF processing and secretion [16] and contribute to efficient protein expression [17]. The O‐glycosylation sites are suggested to be protective toward pro‐protein convertases, and/or act as scaffolds for the localised higher‐order structure in the pro‐part of proNGF [9, 18].

The two alternative spliced pre‐isoforms of proNGF, named transcripts A and B of 34 and 27 kDa respectively are those presenting the signal peptides to entry in the endoplasmic reticulum. The signal peptides are removed to yield proNGF species of 32 and 25 kDa, which are then processed to mature form by furin or other matrix metalloprotease [19]. Fahnestock *et al*. demonstrated that proNGF is the highest NGF form in human, rat, and mouse brains, and several proNGF fragments between 30 and 22 kDa can be detected in brain, as well as in the submandibular and thyroid glands [20, 21]. Different proNGF forms from 50 to 25 kDa are described in the testis and the ovary [22, 23], as well as in the intestine [24].

Shorter fragments derived from the pdNGF have been also found. Dicou *et al*., using an antibody able to recognise specific sequences of NGF prodomain, demonstrated short forms of proNGF, which will be herein indicated as prodomain NGF peptides (pdNGFpeps), in brain, liver and intestine [25], and subsequently that the related synthetic peptides might exert biological function when added to neurons in culture [26].

More recently, it was demonstrated that the sequence of NGF prodomain (spanned residues 19–121 of mouse β‐proNGF) has *per se* the ability to induce growth cone collapse, and it can interact with mature NGF, and p75NTR [26, 27, 28], suggesting a functional independence of NGF prodomain region.

These findings suggest that, besides NGF and proNGF, the pdNGFpeps generated during the endogenous proNGF processing might affect target activity and functions, and therefore that the different expression of proNGF forms in tissues might also have a physiological and/or pathological value.

Dysmetabolism, stress, and inflammation have showed to alter the NGF expression [29] and, like in brain and retina [30, 31, 32], an unbalance of NGF and proNGF expression has been recently proposed as a risk factor for peripheral diseases, also associated with inflammation [33, 34]. There is currently no data on proNGF processing including the pdNGFpeps, in the central nervous system or peripheral tissues, and neither it is known whether inflammatory conditions might affect the relative expression of the pdNGFpeps.

To cover these questions, the present study was aimed to investigate the proNGF processing and identify the expression levels of pdNGFpeps in Cerebellar Cortex, Spinal Cord and peripheral organs of adult rats, as well as to evaluate the effects of systemic inflammation induced by Lipopolysaccharides (LPS) intraperitoneal injection. Different commercial antibodies were primarily tested by WB analysis, and the most efficient to detect endogenous pdNGF fragments was used to analyse the NGF processing, and eventually the presence of pdNGFpeps in rat adult organs.

Further, to validate the bioactivity of the pdNGFpeps, pools of peptides corresponding to the prodomain of NGF, derived from the enzymatic cleavage of recombinant human proNGF molecule were tested *in vitro* by using primary neurons.

Our study confirms that the proNGF is the highest NGF form expressed in rat tissues and that short fragments corresponding to the prodomain of proNGF are detectable, and differently expressed in the tested organs. More, the expression of pdNGFpeps is subjected to change during inflammation suggesting that proNGF processing might be correlated with the organ functions and response to insults and/or injury.

Finally, our study demonstrates that pdNGFpeps have biological activity when added to primary neurons in culture, showing that only the short peptides from the residues 1–60, but not those corresponding to the 70–110 of the prodomain sequences, exert apoptotic and/or anti‐proliferative activity. These findings corroborate the idea that the prodomain of proNGF has its own bioactivity, and therefore that different NGF pattern processing in targets might reflect the spectrum of action of NGF in physiological and pathological conditions.

## Materials and methods

The present study includes experiments *in vivo* on adult male rats to investigate the expression of proNGF in different organs, and *in vitro* on primary cells obtained by rat embryos to evaluate the potential bioactivity of pdNGFpeps. All the animals used in this study were handled in compliance with the National (D.Lgs26/2014) and European Union legislation guidelines for animal welfare (2010/63/EU), and approved by the Italian Ministry of Health (Authorization no. 668/2016‐PR). The principles of the 3Rs as defined by Russell and Burch in their book “The Principles of Humane Experimental Technique” were also taken into consideration.

### NGF, proNGF and pdNGFpeps

The human recombinant NGF and proNGF produced in *Escherichia coli* were obtained by Dompè Farmaceutici Spa and used to screen the anti‐NGF and anti‐proNGF antibodies by Western blot (WB) and dot blot.

To obtain the pool of peptides derived from the NGF prodomain, proNGF was incubated overnight at room temperature with trypsin, and the mixture was separated by Cation Exchange (CEX) gel chromatography; the factions were selected by reverse phase (RP) UPLC and then by Mass Spectrometry to verify the removal of mature NGF.

The peptide pools C1 (from 70 to 104 aa of proNGF sequence) and C6 (from 1 to 66 aa of proNGF sequence) were selected, their volumes were measured, and the protein content was determined by bicinchoninic acid assay (BCA). Each pool was then lyophilized. The C1 and C6 pools were rehydrated at 100 μg·mL^−1^ to be used in the dot blot assay and *in vitro* experiments.

#### Dot blot assay

Antibodies against proNGF (Alomone Labs, Jerusalem, Israel ANT‐005; 1 : 1000, and Biosensis, Thebarton, South Australia M‐1778‐B; 1 : 1000, S‐080; 1 : 1000), and NGF (Abcam, Cambridge, UK, ab6199, 1 : 1000) were preliminary used to evaluate their reactivity and selectivity to recognise NGF prodomain and/or its mature form by dot blot. Firstly, a volume of 0.5–1 μL of NGF, proNGF, C1 and C6 was separately pipetted on nitrocellulose membranes, and then, after their complete absorption, the membranes were blocked in non‐fat dried milk 5% prepared in TBS‐T (10 mm Tris, pH 7.4, 100 mm NaCl, and 0.1% Tween‐20), for 30 min to 1 h on a rotating shaker at room temperature (RT). After, the membranes were washed with TBS‐T three times 5–8 min each and incubated with the primary antibodies previously described for 30 min to 1 h on a rotating shaker. Then, the membranes were washed with TBS‐T, three times 5–8 min each, and incubated for 40 min on a rotating shaker at RT with the secondary antibodies Goat Anti‐Rabbit and anti‐Mouse Conjugated to Horseradish Peroxidase (Bio‐Rad, Milan, Italy, #1706515 and #1706516, respectively; 1 : 2000). The membranes were then washed with TBS‐T three times 10 min each and the Enhanced Chemiluminescence (ECL) was used to detect the signal using the iBright™ CL1500 Imaging System (ThermoFisher Scientific, Waltham, MA, USA).

### *In vivo* study

Male Sprague Dawley rats (body weight 220–250 g) purchased from Charles River (Charles River Laboratories Italia s.r.l., Calco, Italy) were used in this study. Animals were kept two per cage under controlled temperature, humidity, and illumination, with food and water *ad libitum*, and a 12 h light‐dark cycle. The animal conditions were checked by a veterinary during the experiments. The rats were randomly divided into two groups: the untreated group named as Control (CTRL) and the Lipopolysaccharides (LPS) group which received a single intraperitoneal injection of saline or Lipopolysaccharides (LPS, 5mg·kg^−1^ b.w.) respectively. Animals from both groups were later killed 48 h after the injection by guillotine. The brain was immediately removed from the skull to dissect the cortex. The Spinal Cord and the organs, such as the Testicles, Intestine, Spleen, Stomach, Kidney, Heart and Liver were also dissected, and washed in saline after removing fat and connective tissue. All the samples were coded and kept at −80 °C for subsequent analysis.

#### Tissue lysate preparation

The collected samples were homogenised by ultrasonication in RIPA buffer (50 mm trisHCl, pH 7.4; 150 mm NaCl; 5 mm EDTA; 1% Triton X−100; 0.1% SDS; 0.5% sodium deoxycholate) supplemented with inhibitors: PhenylMethylSulfonyl Fluoride (PMSF), Leupeptin, Protease and Phosphatase inhibitor cocktails (P8340 and P5726, respectively), and was left at +4 °C on a rotator for 2 h to allow the complete tissue disaggregation and cell lysis. It was then centrifuged at 17005 ***g*** for 30 min at +4 °C, to remove tissue debris. The supernatants were used for total protein concentration measured by the Bio‐Rad assay. All lysate samples were used for WB as described below.

#### Western blot assay

The previously described prepared lysates were used for WB analysis. The lysates (20–50 μg) were dissolved in loading buffer and heated, they were then resolved on 10–15% SDS/PAGE at 30 mA (constant current) for about 120 min. Proteins were transferred onto nitrocellulose membrane using trans‐blot turbo transfer system (Bio‐Rad Laboratories) for 10 min at RT. The nitrocellulose membranes were blocked for 1 h at RT either with 3% Bovine Serum Albumin (BSA) or 5% non‐fat dried milk in TBS‐T (10 mm Tris, pH 7.4, 100 mm NaCl, and 0.1% Tween20). Then, the membranes were washed three times for 10 min each at RT in TBS‐T and were incubated at +4 °C overnight with the primary antibodies dissolved in TBS‐T or BSA. Afterward, the membranes were incubated for 1 h with Goat Anti‐Rabbit or anti‐Mouse Conjugated to Horseradish Peroxidase (Bio‐Rad, #1706515 and #1706516, respectively; 1 : 2000). Antibody against β‐Actin (Santa Cruz Biotechnology, Dallas, TX, USA; sc‐47778 HRP‐conjugated, 1 : 5000) was used as housekeeping protein. Clarity Western enhanced chemoluminescence detection ECL (Bio‐Rad) was used for Immunoblot analysis and image acquisition was performed by iBright™ (CL1500 Imaging System; ThermoFisher Scientific). Relative levels of immunoreactivity were determined using densitometry and the software imagej (National Institutes of Health, Bethesda, MD, USA) for Windows 10. Values are expressed as arbitrary OD units, and the data are presented as means ± Standard Deviation (SD).

### Effects of proNGF and pdNGFpeps *in vitro*

#### Primary neurons dissociation and analysis

Hippocampal neurons were prepared from embryonic day 17–18 (E17–18) embryos from timed pregnant Wistar rats (purchased from Charles River). The embryonic hippocampus was dissected out in Hepes‐buffered Hanks' balanced salt solution and dissociated via trypsin/EDTA treatment. Cells were seeded at 5 × 104 cells on coverslips on glass coverslips in 24‐well plates (BD Falcon, New York, NY, USA; 351147) for immunostaining and 5 × 105 in 3.5 cm poly(d/l‐lysine) pre‐coated dishes for biochemistry. Cells were cultured in neurobasal medium (NB) supplemented with B‐27 and glutamax until maturity (DIV7). Half of the medium was changed every 2–3 days. Hippocampal neurons (DIV7) were incubated for 1 h with (100 ng·mL^−1^) of NGF, proNGF, C1 and C6. The number of neurons activated towards the proapoptotic cascade was evaluated by means of anti‐phosphorylated c‐Jun N‐terminal kinase (pJNK) staining. Untreated neurons were used as control (CTR).

##### Immunofluorescence labelling and microscopy

Primary neuron cultures were fixed for 20 min in PBS containing 4% paraformaldehyde, permeabilized with PBS plus 0.3% Tween, and quenched by ammonium chloride (50 mm, 30′, RT). A specific staining by the secondary antibody was blocked by incubation with normal donkey serum (10%, 1 h, RT). The overnight incubation (+4 °C) with primary antibodies was followed by the appropriate secondary antibodies (1 : 2000, 1 h, RT). Rabbit anti‐pJNK (Thr183/Tyr185, CST, Danvers, MA, USA, 9251; 1 : 100, overnight, +4 °C) followed by incubation with donkey anti‐rabbit Alexa‐488 antibody (Life Technologies, Carlsbad, CA, USA; 1 : 1000, 1 h, RT). Nuclei were counterstained with 4',6‐diamidino‐2‐phenylindole (DAPI; Life Technologies), mounted on coverslips with Prolong Gold Antifade Mounting (Life Technologies) and kept at −20 °C before image analysis.

##### Neuron count

Immunofluorescence was acquired with an epifluorescent microscope (Leica CTR5500; Leica Microsystems, Buccinasco, Italy) equipped with a CCD camera (Leica). Images for direct comparison were collected using the same settings. The number of positive neurons/nuclei over the total number of DAPI stained nuclei per field (field area = 0.366 μm^2^; 20× objective) was measured in 5 different fields per coverslip. The analysis was performed after calibrating for particle pixels' size (50–400 pixels) and applying a fixed threshold over the background. Nuclei were counted both by manual and automated counting methods (imagej software, 1.52t, NIH) with comparable results. Data are expressed as percentage and presented as mean ± Standard error of the mean (SEM).

### Analysis

The statistical analysis was conducted using two‐way ANOVA, with groups (CTRL and LPS), followed by Student's *T*‐test *post‐hoc* or Tukey's *post‐hoc* test analyses using prism (GraphPad Software, Inc., San Diego, CA, USA) or statview (Statview‐SAS, Cary, NC, USA). All data are presented as the mean ± SD, and a *P* value ≤ 0.05 was considered statistically significant.

## Results

### NGF and proNGF detection by commercial antibodies

To detect NGF and proNGF, Abcam antibody (ab6199) against NGF and anti‐proNGF antibodies from Biosensis (M‐1778‐B and S‐080) and Alomone (ANT‐005) were used. Table 1 shows the specifications of the antibodies taken into consideration.

**Table 1:** Commercial antibodies for NGF and proNGF detection.

Antibody code	Description	Immunogen type	Database link	Datasheet link
Ab6199	Rabbit polyclonal to NGF	Mature NGF	P01139	https://www.abcam.com/ngf‐antibody‐bsa‐and‐azide‐free‐ab6199.html
M‐1778‐B	Mouse monoclonal to proNGF	ProNGF	P01138	https://www.biosensis.com/mouse‐monoclonal‐antibody‐prongf‐clone‐bs312‐biotinylated‐1596.html
S‐080	Sheep polyclonal to proNGF	ProNGF	P01138	https://www.biosensis.com/sheep‐antibody‐human‐prongf‐whole‐serum‐436.html
ANT‐005	Rabbit polyclonal to proNGF	ProNGF	P25427	https://www.alomone.com/p/anti‐prongf‐2/ANT‐005

**Fig. 1:**
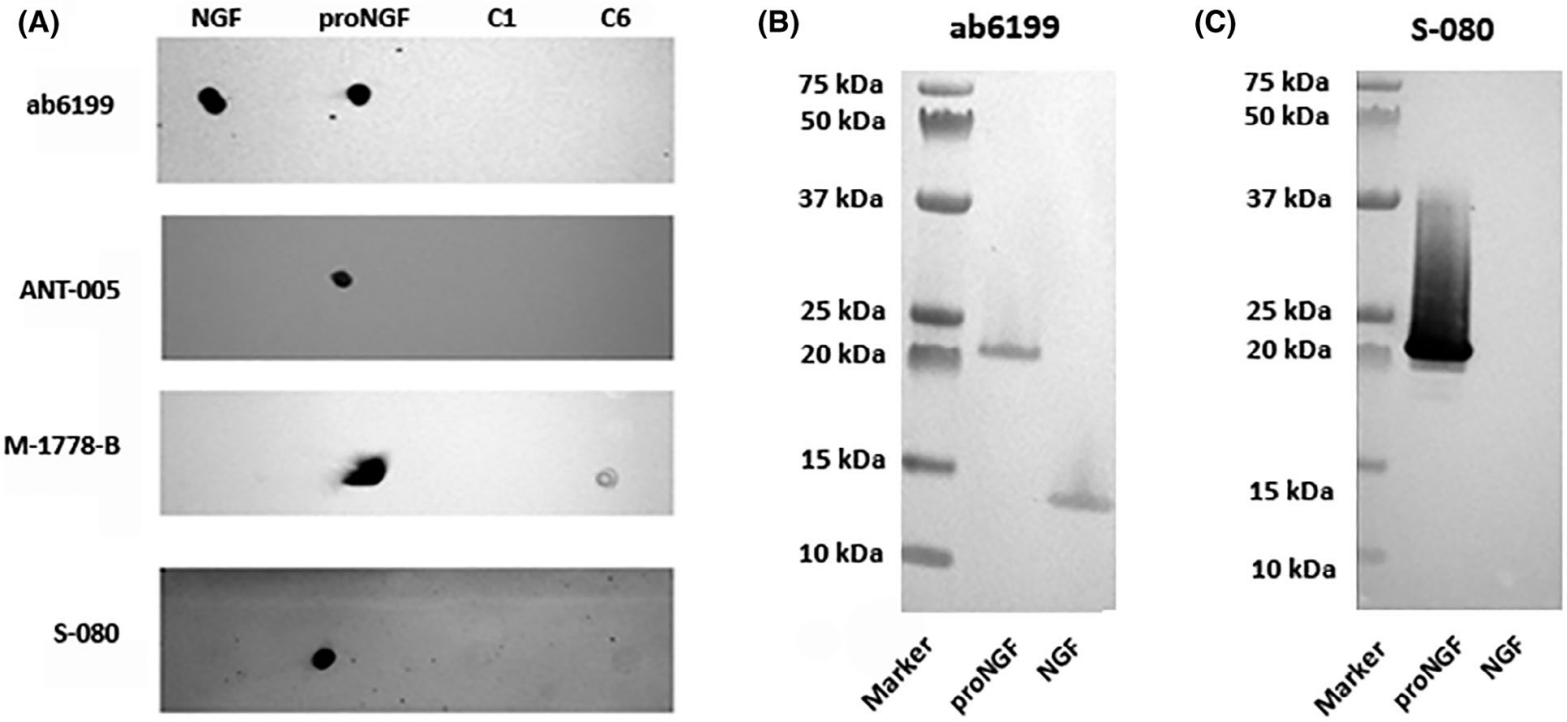
(A–C) Dot blot and western blot for commercial antibody selectivity towards proNGF and/or NGF. (A) Detection of NGF, proNGF, C1 and C6 by using the different antibodies against NGF and proNGF: Abcam ab6199, Alomone ANT‐005 and Biosensis M‐1778‐B or S‐080. (B, C) The representative western blots show the ability and selectivity of (B) Abcam anti‐NGF antibody and (C) Biosensis (M‐1778‐B) anti‐proNGF antibody to detect recombinant NGF and/or proNGF.

The dot blot assay performed using proNGF, NGF, C1 and C6 was used to investigate the different anti‐proNGF or anti‐NGF antibodies affinity/sensibility towards these neurotrophins and the related peptide pools (Fig. 1A). The analysis shows that ab6199 can recognise both NGF and proNGF but not the pools of pdNGFps, ANT‐005 detects only proNGF, while M‐1778‐B is able to detect both proNGF and C6, but not NGF or C1. Further, by using S‐080, it was observed a specific detection of proNGF as well as a slight revealing of C6 (Fig. 1A). Then, we evaluated the sensibility of these antibodies for using recombinant mature NGF (NGF) or its precursor (proNGF) samples, by using the western blot method (Fig. 1B,C). As expected by the type of immunogen used for the antibody preparation, in Fig. 1B, ab6199 has a high affinity for mature NGF but also detects recombinant proNGF showing bands around and below 25 kDa. In Fig. 1C, M‐1778‐B recognises recombinant proNGF, but not mature NGF. Similar results are obtained using another specific antibody against proNGF from Biosensis (S‐080) and Alomone (ANT‐005) data not showed.

### ProNGF western blot analysis in rat organs

**Fig. 2:**
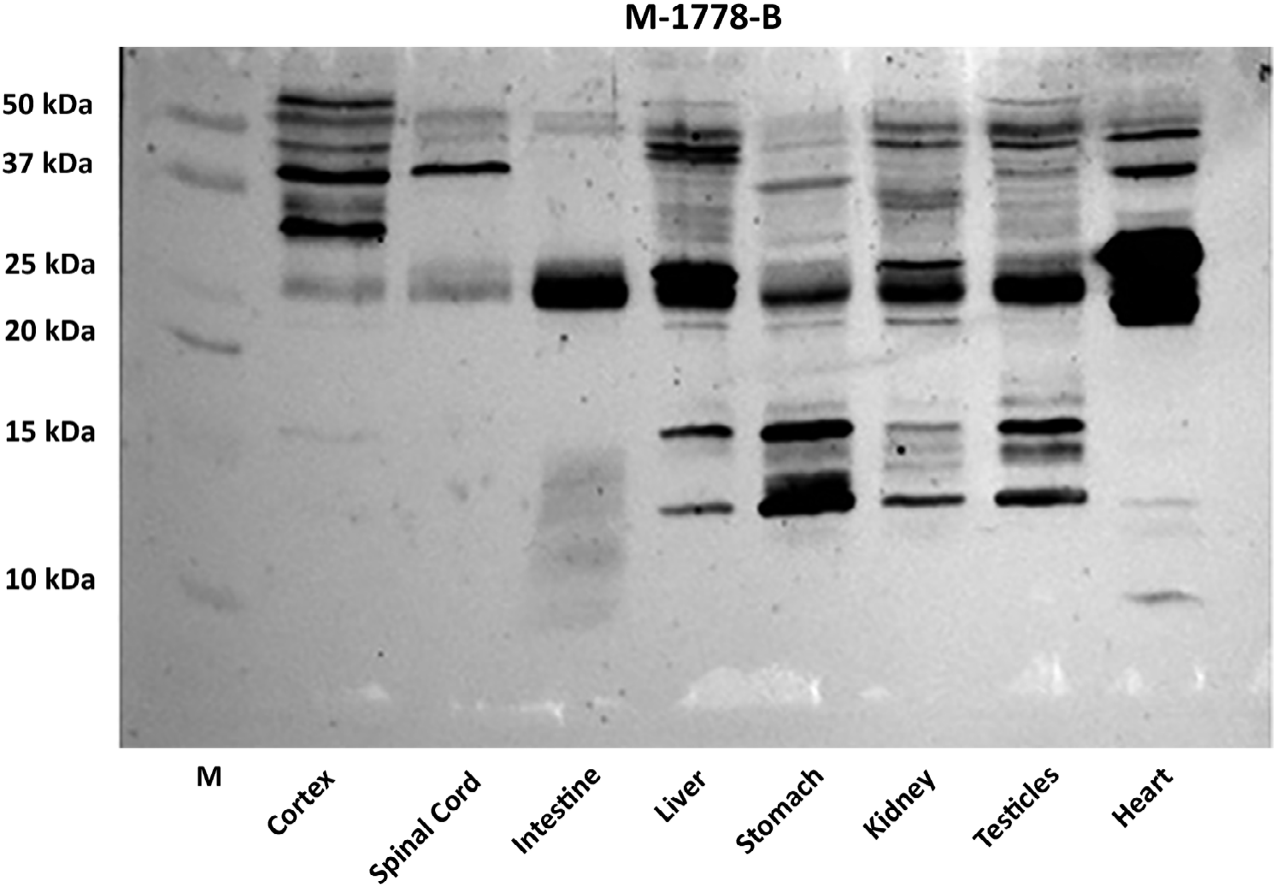
Expression of proNGF forms in different organs of adult male rats detected by Biosensis Antibody M‐1778‐B. Representative western blot of proNGF (Biosensis M‐1778‐B) detection in rat cortex, spinal cord and several peripheral tissues. “M” stands for protein marker.

Based on the results obtained by the antibody screening, the anti‐proNGF antibody by Biosensis (M‐1778‐B) was used to further investigate the proNGF expression, as well as that of its related fragments (Fig. 2). A representative proNGF western Blot for Cerebral Cortex, Spinal Cord, and peripheral organs of adult rats is showed in Fig. 2, by using M‐1778‐B. Compared to Cerebral Cortex and Spinal Cord, where only bands over 25 kDa were detected, bands over and below 15 kDa are observable in almost all the analysed rat organs. A very low expression of these low bands (indicating the presence of the pro‐part) is found in the intestine and heart, while the highest are observable in the stomach and testicles.

### Effects of LPS on the proNGF expression levels

**Fig. 3:**
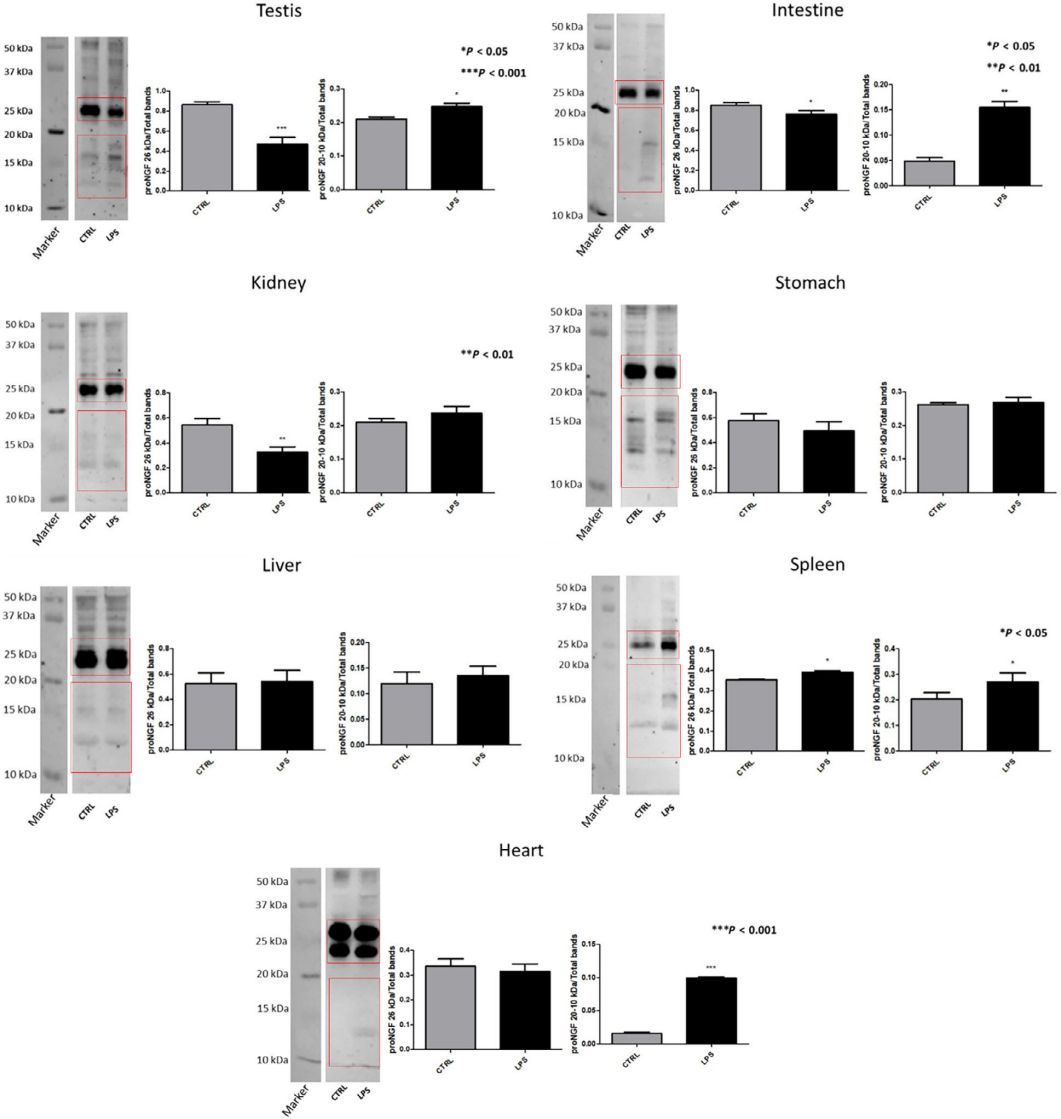
Expression of pdNGFpeps in different organs of adult male rats detected M‐1778‐B. Representative western blots of proNGF detection in rat testis, intestine, kidney, stomach, liver, spleen and heart. CTRL = healthy rats; LPS = LPS challenged rats. The statistical analysis was conducted using a Student's *T*‐test. The number of biologically‐independent replicates is 3. Graph bars report the mean + SD. Statistical significance: *P < 0.5; **P < 0.01; ***P < 0.001.

The expression levels of proNGF were then investigated by WB using M‐1778‐B antibody as it is observable in the representative gels in Fig. 3, a common decrease of proNGF (26 kDa) level is found in almost every tissue after LPS administration. Short proNGF fragments were detected in tissues of both the CTRL and LPS group. The most evident LPS effect was found in the heart and intestine where the pdNGFpeps level was more than three times than in CTRL (significant *P* < 0.001 and *P* < 0.01, respectively). Significant pdNGFpep increase was also observed in the testis and spleen, while no changes in pdNGFpeps were found when analysing kidney, stomach and liver.

### Effects of pdNGF peptide pools C1 and C6 on hippocampal neurons.

**Fig. 4:**
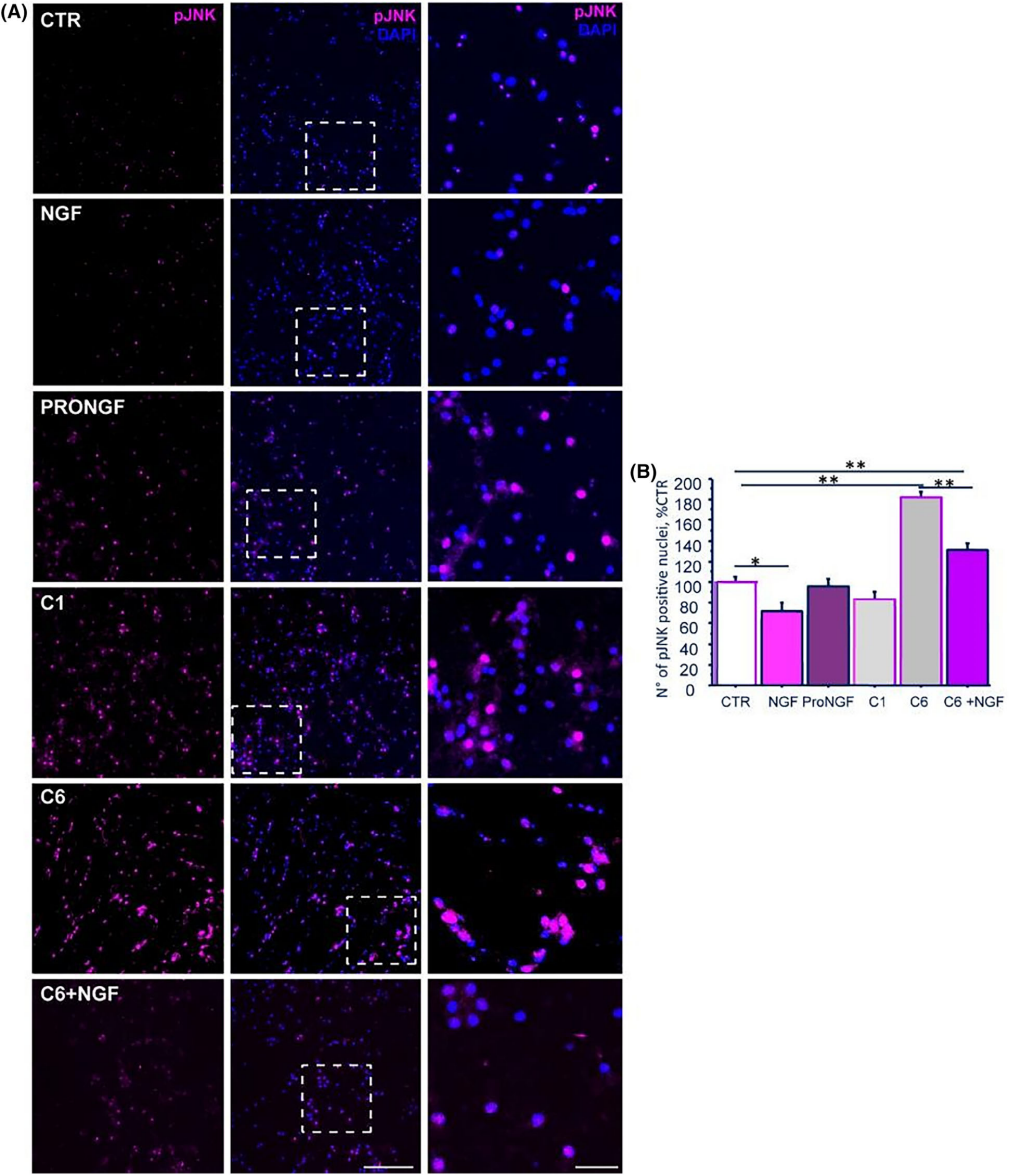
(A, B) Effects of NGF, its unprocessed precursor proNGF, C1 propeptide and C6 propeptide on hippocampal cells (DIV). (A) Representative immunofluorescence for pJNK in hippocampal cells (DIV) treated for 1 h with NGF, its unprocessed precursor proNGF, C1 propeptide and C6 propeptide alone or with NGF. (B) Quantification of the proapoptotic effects of NGF and proNGF peptides on the activation of cells toward the p75/JNK‐driven proapototic pathway. Number of hippocampal neuronal nuclei positive to pJNK. Values represent the number of nuclei expressing pJNK, as percentage of CTR. Graph bars report the mean + SEM. *N* = 5 per experimental group. Reported significance is calculated by Student–Newman test. Statistical significance: *P < 0.5; **P < 0.01. Scale bars: 200 μm; inlet: 50 μm.

Based on the demonstrated ability of proNGF to activate neurons towards the proapoptotic cascade [35, 36, 37], the effect of C1 and C6, and proNGF and NGF treatments were analysed in hippocampal neurons by using the phosphorylation of JNK as apoptotic initiation signalling marker. As shown in Fig. 4A, compared to untreated neurons (CTR), an increase of pJNK expression is observable in hippocampal cells treated with proNGF, and mainly with C6. Only few pJNK positive cells were observable following C1 and NGF. A reduction of pJNK staining was found when NGF was added to cells treated with C6. Noteworthy, the vast majority of the pJNK positive signal is restricted to the cell nuclei, accordingly with a transcriptional role of JNK signalling in our experimental conditions. The count of pJNK expressing (*magenta*), nuclei (*blue*, DAPI) and the related statistical analysis confirm the microscopy observations showing that NGF decreased the number of hippocampal neurons expressing pJNK (72.0 ± 8.4% CTR; *P < 0.05), as expected based on previous observation in the cholinergic neuronal cell type [38], while the C6 fragment induces in turn a substantial increase of pJNK positive nuclei (182.5 ± 4.9% CTR; **P < 0.01) in the hippocampal culture. Further, NGF was able to significantly downregulate JNK activation also when co‐incubated with the C6 propeptide (130.7 ± 6.5% CTR; C6 + NGF vs. C6: **P < 0.01), although not at the level of CTR neurons (C6 + NGF vs. CTR: **P < 0.01). The ProNGF (95.5 ± 7.4% CTR), and the C1 fragment (83.1 ± 7.8% CTR) had no statistically significant effect. ANOVA followed by *t*‐Student *post‐hoc* test, was used to evaluate statistical significance (Fig. 4B).

## Discussion

The expression of proNGF in brain and peripheral tissues [39], and the identification of different active forms derived from its cleavage, including prodomain peptides [25] have opened new questions about the biological role of proNGF processing in mammalian physiopathology. The present study was addressed to investigate the expression levels of proNGF peptides in organs of adult rats with or without a challenge with inflammatory stimuli such as the intraperitoneal (i.p.) LPS injection. Moreover, to verify the hypothesis that the NGF prodomain peptides (pdNGFpeps) might be biologically active, two different pools of prodomain peptides derived from the enzymatic cleavage of the proNGF were tested *in vitro*. To analyse the endogenous proNGF processing, we have first selected commercial antibodies directed against the N‐terminal sequence of the proNGF molecule and tested their ability to detect specific proNGF fragments in tissue extracts. We found that the two anti‐proNGF antibodies produced by Biosensis (code M‐1778‐B and S‐080) recognise recombinant proNGF forms but not the NGF purified peptide, confirming that all signal detected in the tissues extract are imputable to the pro‐part still linked to the mature protein or cleaved during its maturation. The M‐1778‐B antibody also detects the C6 pool of prodomain peptides derived from the enzymatic digestion of the entire precursor molecule (Fig. 1) and confirms its ability to identify the proNGF and its fragments in adult rat tissues. In accordance, several bands corresponding to proNGF were detected by WB analysis in cerebral cortex and spinal cord, as well as in peripheral organs of adult rats by using this antibody (Fig. 2). In our experimental conditions, optic bands at size < 50 and > 25 kDa, with a marked signal around 37kDa—described previously either as the preproNGF‐A [40] or as the glycosylated form of proNGF‐B [41]—were found in the cerebral cortex and spinal cord. These data confirm the observations that the unprocessed proNGF is the major NGF form expressed in central nervous system [39]. However, in line with what has been found by Dicou [20] and Reinshagen [42], our ongoing studies are showing that mature NGF but also proNGF fragments at low molecular weight are detectable in the hippocampus, the retina, and the DRG (unpublished). These data underline the importance of a better understanding of proNGF processing, and eventually identify the pdNGFpep role in the nervous system tissues.

As far as the proNGF expression in periphery is concerned, we found that different proNGF fragments at size lower than 30 kDa are present in the analysed organs. In detail, a strong WB signal at size higher than 25 kDa, corresponding to the transcript B [40] is detectable together with other bands between 20 and 10 kDa which are present in the liver, stomach, kidney, and testis, and scarcely in heart and intestine of healthy rats. These results are consistent with the types of proNGF fragments previously found in adult rat tissues using an antibody against the −71 to −43 sequence of NGF precursor [24, 25] and suggest that NGF processing is organ‐specific. Moreover, the two main bands over and below 15 kDa which we detected in almost all the analysed rat organs might reasonably correspond to the prodomain NGF region derived by the furin cleavage of the Transcript A or B. The evidence that protein fragments of 13, 18 and 22 kDa are detectable following *in vitro* proNGF digestion [43], and that WB analysis of uncleavable proNGF form treated with furin, and the isolated pro region results in fragments of about 17 and 14 kDa respectively [44] support our idea.

Our results on the endogenous NGF processing analysis in inflamed tissues, and our in vitro study using pools of prodomain peptides further suggest a biological function for the pdNGFpeps. Indeed, we demonstrate that a challenge with i.p. LPS, which induces an inflammatory cascade in periphery and brain [45, 46], results in a decrease of proNGF transcript form, but an increase of pdNGFpep levels in testis, stomach, intestine, spleen, and heart. These results corroborate the evidence that inflammation stimulates NGF in peripheral tissues [47], but they also show an accumulation of cleavage‐derived prodomain peptides in the analysed organs. To the best of our knowledge, no other studies investigated the effects of systemic LPS on proNGF processing in organs, but short prodomain peptides were found in serum and synovia fluids of arthritic patients, as well as in the serum of patients with systemic lupus erythematosus (SLE) and thyroiditis [48], supporting a correlation between the presence of pdNGFpeps and inflammation.

Further studies will be necessary to verify whether and how the pdNGFpeps might be involved in inflammation, but since the activation of anti‐ and proinflammatory pathways depends on the balance between NGF/proNGF, and on the relative expression of their receptors [49], it is possible to hypothesise the prodomain peptides acting as ligand of NGF receptors might trigger intracellular pathways which contrast or sustain the action of NGF or proNGF as full molecules. Also, the accumulation of pdNGF might be due to inflammation‐dependent modification of proteases involved in proNGF maturation or NGF degradation. Since it was reported that there exists an activity‐dependent conversion of proNGF to NGF, and a coordinated release and activation of proenzymes and enzyme regulators [50].

The evidence that NGF prodomain peptides have the ability to bind the NGF receptors and/or affect their signallings [24, 51], and that NGF, proNGF wild‐type, and proNGF furin‐cleavage resistant activates different transcriptional programs, which only partially overlap [52] support our hypothesis that the endogenous pdNGF peps are biologically active, and might contribute to the multi NGF actions and effects by acting as receptor ligand.

In line with this, our *in vitro* study demonstrates the ability of prodomain peptide pools derived from the enzymatic cleavage of proNGF to affect neuronal survival when added in cultures. Specifically, we show that the C6 pool induces an increase of JNK activation, and that its proapoptotic effect on neurons is obstacle by the co‐treatment with mature NGF, while no effects of the peptide pools C1 (from 70 to 104 aa of proNGF sequence) was observed. These findings suggest that the proapoptotic ability of proNGF is mainly due to the C‐terminal sequence and not to sequences closer to the mature NGF motive, like those in C1 pool. These findings are in agreement with Yan *et al*. [26] who showed that the pdNGF‐induced apoptosis and the growth cone collapse and indicate that the specific sequences comprised between 1 and 66 aa of proNGF sequence (C6 pool) is crucial to activate JNK death pathway. Since p75NTR and sortilin are known to mediate the proapoptotic effects of proNGF [53], and an involvement of p75NTR is suggested by Yan *et al*. [26] by the findings that the proapoptotic effect of pdNGF is greater in p75^NTR^ overexpressing neurons, it is possible that 1 and 66 aa of proNGF sequence might facilitate the proNGF bindings to p75NTR and/or interact directly with sortilin, as suggested by Feng and collaborators [54].

However, Dicou *et al*. [25] reported that prodomain peptides are able to activate TrkA in PC12 cells; therefore, the possibility that pdNGF might also act through this receptor type cannot be excluded. The involvement of NGF receptors in mediating the effects of pdNGFpeps is currently under investigation in our laboratory.

In conclusion, our study suggests that, like to other growth factors, as VGF [55], the NGF biological activity is not exerted only by the mature NGF and proNGF full‐length molecule, but it could be also due to fragments derived by the precursor processing, including the pdNGFpeps. This hypothesis involves that, together with NGF/proNGF ratio, the relative amount of the different biologically active NGF peptides might contribute to maintain the structural and functional integrity of target tissues, and consequently represent a further marker of insurgence and/or progression of pathological conditions.

Moreover, showing that specific sequence of the prodomain region of the proNGF molecule might exert apoptotic function which is contrasted by the NGF, our study enlarges the knowledge on the potential endogenous ligands of the NGF receptors and suggests new molecular targets to drug design studies.

## Conflict of interest

The authors declare no conflict of interest.

## Authors contributions

PT conceived and designed the study; PT coordinated the experimental plan; EF and PR performed the *in vivo* treatment; LDS, DR, FC and MA developed peptides and protein; MAM, EF and PR did the sample collection; MAM, EF and PR carried out sample preparation and performed western blot; MAM and VT analysed the data and performed statistical analysis; VT performed immunofluorescence and microscopy images collection; MAM, EF, PT and VT were involved in the interpretation of the data; MAM, EF and PT wrote the manuscript; PT, MAM and EF, critically discussed the results, PT, MAM, EF, PR, VT, FC, MA revised the manuscript, all the authors approved its final version and qualify for authorship. All authors read and agreed to the published version of the manuscript.

## Data Availability

The data that support the findings of this study are available from the corresponding authors: paola.tirassa@cnr.it; elena.fico@ibbc.cnr.it upon reasonable request.
